# Immunological mechanisms of autoimmune gastritis

**DOI:** 10.1007/s10238-026-02080-z

**Published:** 2026-02-11

**Authors:** Jiarun Qian, Zhen Hu, Zihan Xu, Shiqing Yuan, Jiaying Zhao, Hongli Shi, Xiaoyun Wang

**Affiliations:** 1 Division of Gastroenterology, Jiangnan University Medical Center (Wuxi No. 2 People’s Hospital), Wuxi, 214000 Jiangsu China; 2 School of wuxi Medicine, Jiangnan University, Wuxi, 214000 Jiangsu China

**Keywords:** Autoimmune gastritis, Immunological mechanisms, CD4⁺ t cells, B cells, H⁺/K⁺-ATPase, Animal models.

## Abstract

Autoimmune gastritis (AIG) is a chronic disease characterized by specific immune damage to the gastric mucosa. Previous studies have mostly focused on the single immune pathway mainly mediated by T cells, but the synergistic role of humoral immunity in disease progression cannot be ignored. This article systematically reviews the immunological mechanism of AIG, and analyzes the inflammatory cascade immune mechanism centered on the self-attack of gastric parietal cells mediated by CD4^+^ T, with the pro-inflammatory roles of Th1/Th17 cells and defective suppressive function of Tregs as a supplement. This article emphasizes the imbalance between humoral and cellular immunity, including the pathogenic potential of autoantibodies and the synergistic role of T-B cells in promoting inflammation. Furthermore, while existing animal models (including genetic modification, lymphopenic, and non-lymphopenic models) can replicate features of human AIG such as gastric gland atrophy, they exhibit significant limitations regarding the mechanism of T-B cell collaboration, differences in cancer risk, and species specificity. This article systematically clarifies that AIG results from an imbalance between cellular and humoral immunity, providing a theoretical basis for targeted immunotherapy strategies.

## Introduction

AIG is a chronic non-self-limiting disease characterized by specific immune damage to the gastric mucosa [1]. The global prevalence of AIG is approximately 0.5% to 4.5% [2–4]. The characteristic pathological alterations in AIG involve irreversible glandular atrophy in the gastric fundus and body, accompanied by progressive destruction of oxyntic glands and hyperplasia of enterochromaffin-like (ECL) cells [5, 6]. The disease often progresses insidiously in its early stages, frequently without significant clinical symptoms [7]. As substantial loss of parietal cells leads to severe hypochlorhydria and intrinsic factor deficiency, subsequent complications may arise. These include pernicious anemia (PA), Vitamin B12 deficiency-associated neuropathy and Type I gastric neuroendocrine tumors [8]. Current therapeutic strategies for AIG remain confined to symptom-directed replacement therapy (primarily through vitamin B₁₂ supplementation), with no curative interventions targeting the underlying autoimmune etiology [1]. The core challenge underlying this situation is that the disease’s pathogenesis has not been fully elucidated, hindering the development of targeted, etiology-based interventions.

**Fig. 1:**
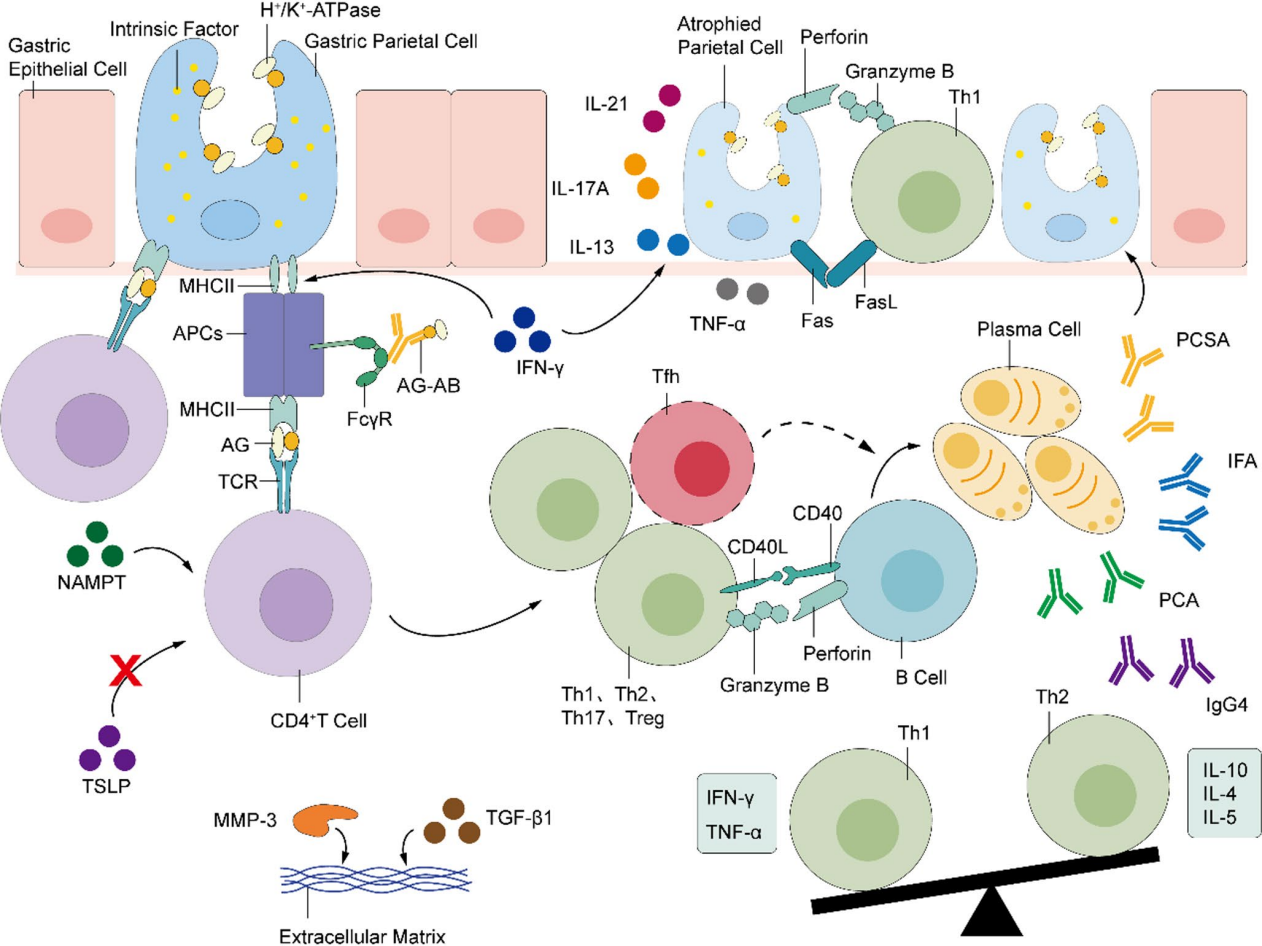
An Overview of AIG Immune Mechanisms: (1) Activated autoreactive CD4^+^ T cells are aberrantly activated upon T-cell receptor (TCR) recognition of antigenic epitopes—derived from parietal cell H⁺/K⁺-ATPase or intrinsic factor and processed by antigen-presenting cells (APCs)—leading to massive cytokine secretion and immune responses. (2) Th1 cells secrete IFN-γ, which upregulates MHC II expression on gastric epithelial cells, while simultaneously mediating direct cytotoxicity via activation of the Fas-FasL apoptotic pathway and granzyme B and perforin. (3) Gastric epithelial cells act as non-classical APCs to present antigens and activate CD4⁺ T cells. (4) Th1/Th2 Cell Imbalance.5) Complement-dependent IgG antibody (PCSA) induces cytotoxic effects. 6) Antigen-antibody complexes enhance antigen presentation by engaging FcγR. 7) Tfh activate autoreactive B cells, promoting their differentiation into plasma cells. 8) The synergistic high expression of MMP-3 and TGF-β1 exacerbates tissue damage via extracellular matrix degradation pathways. Abbreviations: IL-4/5/17A/21/10: Interleukin-4/5/17A/21/10; Th1/2/17: T helper 1/2/17 cell; Treg: Regulatory T cell; Tfh: follicular helper T cell; IFN-γ: Interferon-gamma; TNF-α: Tumor necrosis factor-alpha; MMP-3: Matrix metalloproteinase-3; TGF-β1: transforming growth factor-beta 1; TSLP: Thymic stromal lymphopoietin; NAMPT: Nicotinamide phosphoribosyltransferase; PCA: Parietal cell antibody; IFA: Intrinsic factor antibody; FcγR: Fc-gamma receptors. MHC II: major histocompatibility complex class II molecule

Currently, the core mechanisms of the autoimmune attack in AIG are not fully elucidated. The gastric mucosal immune microenvironment in AIG involves the infiltration of various immune cells, including CD4^+^/CD8^+^ T cells, B cells, plasma cells, and macrophages [9, 10]. Existing research has predominantly focused on single immune pathways, mainly T cell-mediated cellular immunity. However, it is crucial to recognize that humoral and cellular immunity exhibit a synergistic dysregulation in AIG pathogenesis. Accordingly, we summarize the immunopathogenesis of AIG as a immune network (Fig. 1). This framework emphasizes that CD4⁺ T lymphocyte-mediated immune dysregulation serves as the central driver of the disease, where the release of pro-inflammatory cytokines by T helper 1 cells/ T helper 17 cells (Th1/Th17) and functional deficiency of regulatory T cells (Tregs) collectively drive chronic inflammation. Concurrently, humoral immunity contributes to gastric mucosal damage through autoantibody production and complement activation, while T-B cell collaboration not only participates in tissue injury but also enhances antigen presentation via immune complexes, thereby amplifying the T cell response. Furthermore, innate immune cells such as dendritic cells within the gastric mucosal microenvironment, along with key cytokines (e.g., IFN-γ, IL-17, NAMPT), augment the intensity and persistence of the immune reaction. This regulatory network plays a pivotal role in critical processes including autoantigen presentation, inflammatory cascade amplification, and tissue damage [11–13].

Beyond the unclear immune mechanisms, clinical translation requires the development of experimental models that can recapitulate the disease pathogenesis. Currently, experimental models for AIG are limited. Existing animal models — including transgenic, lymphopenic, and non-lymphopenic models — can replicate some features of human AIG, such as gastric gland atrophy. However, they possess significant limitations regarding the mechanisms of T-B cell collaboration, differences in carcinogenesis risk, and species specificity. Therefore, this article also systematically reviews the experimental models of AIG, with the goal of contributing to a better understanding of the disease’s pathogenesis and advancing clinical treatment strategies for AIG.

## The immune microenvironment in AIG

### Cellular immunity

#### Self-Antigen-Driven T cell activation and antigen presentation

The T cell activation process involves antigen presentation, MHC molecule-dependent immune recognition, and key cytokine regulation. H^+^/K^+^-ATPase serves as the core autoantigen in AIG. Its α and β subunits can be processed by local antigen-presenting cells (APCs)into antigenic peptides and presented to the T-cell receptor (TCR) via MHC class II molecules (e.g., HLA-DR) driving polyclonal activation and expansion of T cells [14–16]. Activated autoreactive CD4^+^ T cells can upregulate MHC class II molecule expression on gastric epithelial cells via IFN-γ, thereby enhancing the efficiency of autoantigen presentation. This establishes a positive feedback loop: enhanced antigen presentation efficiency — intensified T cell activation — increased release of inflammatory factors. Ultimately, this cycle leads to the aggravated progressive destruction of the gastric mucosa [9, 17, 18].

APCs provide the basis for T cell recognition and activation by efficiently presenting antigens. However, in the context of autoimmune gastritis, direct experimental evidence regarding the mechanistic role of APCs remains lacking [19]. Dendritic cells (DCs) represent the major professional APCs in the gastric mucosa and play a central role in T cell activation [20, 21]. Nevertheless, Bourges et al. found in a model of acute inflammation that migratory dendritic cells from gastric lymph nodes, although capable of presenting gastric H⁺/K⁺-ATPase antigenic peptides, maintained peripheral tolerance to self-antigens. To date, no direct evidence has demonstrated that prolonged chronic inflammation disrupts the self-antigen tolerance of DCs, thereby leading to the development of AIG [22]. Furthermore, studies have also indicated that gastric epithelial cells can function as non-classical APCs by expressing co-stimulatory molecules B7-1 (CD80) or B7-2 (CD86) during inflammation and infection, thereby activating CD4⁺ T cells to participate in the immune response [23, 24]. Recent studies have revealed that human innate lymphoid cells (ILCs) can function as “non-classical” antigen-presenting cells under the regulation of pro-inflammatory cytokines such as IL-1β and IL-18, thereby activating cellular immune responses [25–27]. We therefore speculate that during the presentation of autoantigens in AIG, ILCs, epithelial cells, and DCs interact and influence one another, collectively forming a complex immunoregulatory network rather than being dominated by a single cell type. This provides a novel perspective for further exploring the gastric mucosal immune microenvironment under the regulation of APCs in AIG.

#### Functional imbalance of T cell subsets and underlying mechanisms

##### Th1

Research has found that the Th1-type immune response predominates during the progression of AIG. Katakai et al. described the chronically inflamed lymphoid tissue induced in the gastric mucosa of a murine Experimental Autoimmune Gastritis (EAG) model as Th1-biased tertiary lymphoid tissue. These ectopic lymphoid structures perpetuate disease progression by sustaining the survival of autoreactive lymphocytes [28].

Studies have found that these Th1 mediate damage to gastric parietal cells primarily through a dual mechanism: (1) approximately 85% of Th1 activate the Fas-FasL apoptosis pathway [29]; (2) a smaller subset of Th1 mediates direct cytotoxic effects via granzyme B and perforin [16]. Despite the general association of cytotoxicity with CD8^+^ T cells, Marshall et al. identified Th1 as the principal mediators of target cell damage in AIG. Their key evidence came from in vitro clonal analysis of T cells from gastric mucosal infiltrates, which revealed no significant reactivity of CD8^+^ T cells to H^+^/K^+^-ATPase [16]. Thus, CD8^+^ T cells likely contribute to immunopathology at a downstream stage of the effector response.

TNF-α and IFN-γ are the primary cytokines secreted by Th1. Research has identified that IFN-γ, secreted by Th1, plays a critical role in the initial stages of AIG. In the lymphopenic EAG model (NTX) model, antigen stimulation significantly enhanced IFN-γ secretion by mouse splenic T cells. Furthermore, a single IFN-γ blockade was found to reduce the disease incidence rate from 42% to 5% [17]. The above study confirms that IFN-γ secreted by Th1 cells has a dual role in both the initiation and progression of the disease: it both enhances the efficiency of antigen presentation and serves as a key mediator of the Th1 inflammatory response.

##### Th2

In the immune imbalance of AIG, the prevailing view holds that Th2 cells play a role in inhibiting disease progression. From the perspective of T-cell subset regulation, the balance between Th1 and Th2 immunity is crucial for maintaining gastric mucosal homeostasis. Th2 secrete inhibitory cytokines such as IL-4, IL-5, and IL-10, which effectively suppress the proliferation and function of Th1, thereby alleviating the pro-inflammatory environment dominated by Th1-type responses [30, 31]. This perspective is supported by the work of Katakai et al., who revealed the differential spatial distribution of Th1 and Th2 cells in vivo. In the NTX-induced EAG model, Th1 cells predominantly infiltrated gastric mucosal lesions to drive damage, whereas Th2 cells were more localized to draining lymph nodes and the spleen. Further investigation revealed that Th1 possess a superior ability for trans-endothelial migration compared to Th2, enabling their differential localization and coexistence within the organism [32]. Based on these spatial distribution patterns, it is postulated that Th2 likely exert systemic immunomodulatory and anti-inflammatory effects by regulating T-cell responses within secondary lymphoid organs, rather than directly participating in the local inflammatory response in the gastric mucosa.

##### Th17

Recent research has elucidated the pro-inflammatory role of Th17 in AIG. Gastric mucosa-specific analyses revealed significantly upregulated expression of IL-17 A and IL-17 F in CD4^+^ T cells from AIG patients following H^+^/K^+^-ATPase stimulation, leading to the further discovery of locally activated, H^+^/K^+^-ATPase-specific Th17 within the AIG gastric mucosa [33]. Troilo et al., studying AIG patients with concomitant PA found that 94% of intrinsic factor-specific T cell clones exhibited a Th17 (53%), Th1 (26%), or Th17/Th1 mixed phenotype (17%), with the majority demonstrating cytotoxicity against gastric epithelial cells, suggesting that Th1 and Th17 collectively drive the inflammation [34].

The primary cytokines secreted by Th17 are IL-17 and IL-21, which have shown participate in various gastric inflammatory processes and even gastric carcinogenesis [35]. Among these, IL-17 A acts as a key pro-inflammatory factor. It can bind to the IL-17RA receptor on parietal cells, activating the caspase-dependent apoptotic pathway. This mechanism drives parietal cell destruction and the formation of spasmolytic polypeptide-expressing metaplasia (SPEM) in the TXA23 mouse model [36, 37]. SPEM has been confirmed as a significant precancerous lesion for gastric adenocarcinoma. Therefore, these findings provide crucial molecular evidence for understanding the mechanisms of AIG-associated gastric cancer development.

##### Treg

As central regulators of immune tolerance, Tregs can suppress autoimmune responses by inhibiting effector T cell activation and autoantibody production. This is achieved through mechanisms including Foxp3 transcription factor-driven gene regulation, secretion of inhibitory cytokines (such as IL-10 and TGF-β), and cell contact-dependent mechanisms reliant on CTLA-4/LAG-3 [38].In AIG, numerical deficiency and functional impairment of Tregs constitute a key immunopathological basis. This understanding is primarily derived from animal models. For instance, Harakal et al. demonstrated that transient depletion of Tregs via diphtheria toxin (DT) in C57BL/6 DEREG mice can induce AIG and render effector T cells resistant to suppression [39]. Similarly, using genetic engineering to cause delayed maturation of Tregs is another established pathogenic mechanism for constructing EAG models. However, within the current framework of AIG pathogenesis, direct research data on the role of Tregs from human AIG patients remain remarkably limited [40]. This paucity of human evidence necessitates caution when directly extrapolating mechanisms of Tregs deficiency or dysfunction from mouse models to human AIG. Future studies should prioritize the direct assessment of Treg number, phenotype, and function through analysis of gastric mucosal tissues and peripheral blood samples from AIG patients to address this critical gap in knowledge.

### Humoral immunity

#### Autoantibodies

Humoral immunity participates in the progression of AIG through mechanisms including autoantibody production, complement activation, and immune complex formation. However, its role varies significantly across different disease stages. Based on current evidence, autoantibodies are not considered the primary initiators of AIG [41]. but rather are more likely to function as diagnostic markers, immune amplifiers, and contributors to disease chronicity.

##### Diagnostic markers

Anti-parietal cell antibodies (PCA) and anti-intrinsic factor antibodies (IFA) are the core serological markers of AIG, holding significant value for screening and diagnosis. PCA (primarily targeting the β-subunit of H⁺/K⁺-ATPase) is positive in up to 90% of AIG patients and can appear even before tissue atrophy, suggesting its role as a marker of early immune activation. IFA, in contrast, is closely associated with vitamin B₁₂ malabsorption and pernicious anemia [6, 19]. Research has found that the average PCA titer in patients with advanced AIG is significantly lower than in those with early or active disease [42]. We posit that this phenomenon may be directly related to the gradual depletion of autoantigens during disease progression, indicating that dynamic changes in antibody titers can reflect the evolution of the in vivo immune activation status.

##### Immune amplification and tissue damage

Although adoptive transfer experiments confirm that T cell-mediated immunity constitutes the core driver of AIG [41], autoantibodies can still markedly amplify inflammation and tissue damage through multiple mechanisms—an effect that may have long been underestimated. Within the spectrum of organ-specific autoimmune diseases, antibodies not only serve as diagnostic markers but also exhibit well-established pro-inflammatory roles, as clearly demonstrated in Hashimoto’s thyroiditis. Significant clinical and immunological links exist between Hashimoto’s thyroiditis and AIG, with both sharing common pathogenic features such as genetic susceptibility, T cell-mediated autoimmunity, and Fas/FasL-mediated target-cell apoptosis [43]. In Hashimoto’s thyroiditis, anti-thyroglobulin antibodies have been shown to induce target-organ injury via complement activation and antibody-dependent cellular cytotoxicity (ADCC) [44]. Similarly, in AIG, early experiments already identified complement-dependent IgG autoantibodies against PCSA in patients’ serum, which can trigger ADCC and exacerbate parietal cell destruction [45]. Moreover, antibodies such as PCA can activate the classical complement pathway, either by forming membrane-attack complexes that directly disrupt the parietal cell membrane or by enhancing phagocytic clearance of target cells through C3b-mediated opsonization [46]. More recently, Saito et al. demonstrated in B-cell-deficient EAG mice that antigen-antibody complexes engage Fcγ receptors (FcγR) on APCs, thereby markedly boosting antigen presentation. This leads to the hypothesis that in AIG, autoantibodies amplify the CD4⁺ T-cell response by enhancing antigen-presentation efficiency [47]. Collectively, this evidence indicates that autoantibodies in AIG are not merely epiphenomena but active and critical regulators that participate in and expand the immunopathological process.

##### Disease chronicity

Histopathological evidence from the gastric mucosa of AIG patients reveals that plasma cell infiltration persists throughout the disease course, with IgG4^+^ plasma cells showing focal aggregation in the gastric mucosa of 37% of patients [48, 49]. From an immunological perspective, IgG4 can undergo Fab-arm exchange to form bispecific antibodies, which may attenuate inflammation by blocking immune-complex formation [50]. A similar phenomenon is observed in Hashimoto’s thyroiditis, where infiltration of IgG4^+^ plasma cells in thyroid tissue correlates significantly with chronic thyroid atrophy and fibrosis [51]. Based on these observations, We speculate that IgG4 antibodies may participate in a negative-feedback loop aimed at counteracting pro-inflammatory responses and could be associated with the development of chronic gastric mucosal atrophy and various forms of epithelial metaplasia in AIG.

#### Collaborative imbalance between humoral and cellular immunity

The immunopathological damage in AIG does not result from a single mechanism but rather stems from the interplay between humoral and cellular immunity. In the early stages of the disease, T cells recognize autoantigens and initiate inflammation. Notably, although the number of B cells in the gastric mucosa of AIG patients does not show a significant increase [9] —which may be related to the simultaneous killing of both parietal cells and B cells by locally infiltrating Th1 via pathways such as Fas-FasL [16]—the widespread infiltration of plasma cells and the focal aggregation of IgG4^+^ plasma cells indicate that B-cell responses within germinal centers, antibody class switching, and plasma cell differentiation remain active. This marks the deep involvement of humoral immunity. Subsequently, plasma cells derived from activated B cells secrete autoantibodies, which further amplify T-cell responses and exacerbate tissue injury through mechanisms such as immune-complex-enhanced antigen presentation, complement activation, and direct cytotoxicity, thereby forming a “T-B cell collaborative circuit”. Accumulating evidence underscores follicular helper T cells (Tfh) as the pivotal orchestrators of T cell-dependent B cell immunity. In autoimmune pathogenesis, dysregulated Tfh activity provides aberrant help to autoreactive B cells within the germinal center. By delivering critical co-stimulatory signals, they subvert the normal checkpoint of clonal deletion, enabling the survival and expansion of self-reactive B cell clones. These B cells, further instructed by Tfh-derived cytokines like IL-21, differentiate into antibody-secreting plasma cells, which in turn are responsible for the pathogenic antibody production that drives inflammation and tissue injury [52–54]. While their role in autoimmune diseases such as primary biliary cirrhosis and primary Sjögren syndrome has been well established [55–57], their function in AIG has not yet been fully elucidated or confirmed, offering an important direction for future research aimed at uncovering this cooperative mechanism.

### Regulatory roles of other cytokines

Beyond the classical cytokines secreted by the immune cells mentioned above, recent studies have identified high expression of Thymic Stromal Lymphopoietin (TSLP) and its receptor IL-7R in the gastric mucosa of AIG patients. Concurrently, the transcriptional level of Nicotinamide Phosphoribosyltransferase (NAMPT) in the AIG gastric mucosa was also found to be elevated compared to normal controls [9].

NAMPT is a critical node linking metabolism and inflammation, and it participates in various disease processes by regulating the production, recruitment, and polarization of myeloid cells [58]. In vitro studies demonstrated that stimulating gastric epithelial cells with eNAMPT (the extracellular form) led to the upregulation of pro-inflammatory factors such as IFN-γ. This suggests that NAMPT may be involved in regulating the gastric mucosal immune landscape and acts as a driver in the pathological process of AIG. Further studies indicate that the coordinated overexpression of Matrix metalloproteinase-3 (MMP-3) and TGF-β1 in the gastric body mucosa may exacerbate tissue damage via MMP-3-mediated degradation of the extracellular matrix [9, 59]. thereby revealing a molecular mechanism underlying AIG progression from the perspective of tissue remodeling.

TSLP, an IL-7-like cytokine, is a key mediator of epithelial-immune crosstalk, primarily secreted by epithelial cells, fibroblasts, and immune cells. It is involved in central tolerance, peripheral homeostasis, and Th2-type immune responses by modulating DC function [60]. In an EAG mouse model, deficiency in the TSLP receptor resulted in aberrant DC activation, promoting increased secretion of IL-12/23p40. This, in turn, drove Th1 differentiation and excessive IFN-γ production, ultimately exacerbating gastric mucosal autoimmune injury. Control mice exhibited significantly lower PCA levels and overall gastritis scores compared to the TSLPR-deficient group, revealing a protective role for TSLP in maintaining gastric mucosal immune homeostasis [61]. Therefore, the elevated TSLP observed in the gastric mucosa of AIG patients might be interpreted as a protective negative feedback mechanism in response to accumulated pathological damage. However, given the significant species-specific differences in TSLP regulation between humans and mice [60], the functional implications of increased TSLP in the gastric mucosa of AIG patients require further experimental clarification.

## AIG animal models and their construction mechanisms

To facilitate in-depth investigation into the pathogenesis of AIG, researchers have developed various animal models [62]. EAG models can recapitulate core features of human AIG, such as gastric mucosal mononuclear cell infiltration, glandular atrophy, and the production of anti-H⁺/K⁺-ATPase antibodies [63, 64]. However, species-specific differences exist. For instance, in mice, intrinsic factor is primarily secreted by chief cells, which differs from humans where it is exclusively produced by parietal cells. Consequently, pathologies related to vitamin B₁₂ malabsorption are difficult to fully replicate in these models [41, 65]. Commonly used models can be categorized into three types: transgenic models, lymphocyte-deficient models, and non-lymphopenic models.

### Transgenic models

The predominant transgenic models currently in use are TCR transgenic mice (Tg), in which the introduction of a gastric parietal-cell-antigen-specific TCR gene leads to excessive activation of effector T cells. Studies have shown that TCR transgenesis can significantly inhibit the developmental process of Tregs [66]. This results in a diminished capacity of Tregs to regulate immune responses. Coupled with the increased ability of effector cells to recognize autoantigens due to the insertion of the specific gene fragment, this ultimately leads to the onset of inflammation.

Among these, the TXA23 transgenic model is the most widely used. This model was constructed by first cloning a pathogenic CD4^+^ T cell clone (A23 clone) derived from a thymectomized mouse-induced EAG model. The TCR cDNA from this clone, which specifically recognizes amino acids 630–641 of the H^+^/K^+^-ATPase α subunit (an I-Ad-restricted epitope), was then targeted for insertion into the host genome [66]. Compared to transgenic models targeting the β subunit, which have a success rate of only 20% [67], the TXA23 model achieves a 100% EAG induction rate due to the higher affinity of its TCR for the antigen.

Another important model is the PC-GMCSF transgenic mouse, which exhibits parietal cell-specific expression of granulocyte-macrophage colony-stimulating factor (GM-CSF). In this model, GM-CSF drives the early accumulation and infiltration of macrophages and dendritic cells within the gastric mucosa. These cells subsequently migrate to draining lymph nodes, where they present autoantigens, thereby activating pathogenic CD4^+^ T cells and initiating an inflammatory cascade reaction [68].

Transgenic models offer high mechanistic precision and relatively comprehensive pathological recapitulation, successfully replicating core features of human AIG. However, such models also exhibit notable limitations. On one hand, the transgenic manipulation may interfere with the natural establishment of immunological tolerance. For instance, TCR transgenesis can suppress Tregs development and excessively enhance effector T-cell activity, which differs from the typically progressive disease course observed in human AIG. On the other hand, these models predominantly focus on simulating the single-dimensional mechanism of T-cell over-activation. While they can recapitulate key phenotypes such as gastric mucosal lymphocytic infiltration and parietal cell destruction, the gastric adenocarcinoma transformation rate they induce is significantly higher than the actual cancer risk in clinical AIG patients [69]. Consequently, these models remain substantially limited in accurately replicating pathological progression and in translational risk assessment.

### Lymphopenic models

The most classic lymphopenic EAG model is constructed in BALB/c mice by surgically removing the thymus from neonatal pups (2–3 days after birth) [70, 71]. In addition to inducingAIG, this model can also lead to concurrent autoimmune diseases such as thyroiditis and oophoritis. CD4^+^ T cells from NTX mice show a significantly enhanced proliferative response to sygeneic APCs. This phenomenon suggests that activated APCs may persistently stimulate T cell expansion and their migration to the gastric mucosa, ultimately causing inflammatory damage [72, 73]. Besides thymectomy, lymphopenic models can also be induced by treatment with cytotoxic drugs [74] or high-dose lymphoid irradiation [75].

The mechanism of broken tolerance in lymphopenic models is relatively well-defined. They induce T cell deficiency via thymectomy or cytotoxic drugs which leads to the expansion of autoreactive CD4⁺ T cells, consistent with the autoimmune origin of AIG. However, the pathology in this model exhibits strong non-specificity, as the mice often develop systemic autoimmune abnormalities, with gastric lesions being just one manifestation, making it difficult to isolate AIG-specific mechanisms. Furthermore, the model’s procedures are complex and raise significant ethical concerns. Neonatal thymectomy is a technically challenging procedure with high postoperative mortality. Additionally, lymphoid irradiation or cytotoxic drug use may introduce non-targeted toxicities.

### Non-Lymphopenic models

Early studies utilized adult (6-week-old) BALB/c mice and induced the EAG model through intermittent subcutaneous injections of murine gastric mucosal H^+^/K^+^-ATPase combined with Complete Freund’s Adjuvant. However, EAG models induced this way exhibited a decline in autoantibody titers and gastric mucosal regeneration after immunization ceased, suggesting the inflammatory response was reversible [76]. Subsequently, researchers successfully established a persistent EAG model in neonatal BALB/c mice using intraperitoneal injection of murine gastric mucosal antigen alone. Studies found that the immune system of neonatal mice is more susceptible to activating autoreactive T cells, which is hypothesized to be related to the immaturity of Tregs at this stage [77, 78]. In recent advancements, Beduleva’s team successfully established an EAG model in rats by immunizing them with homologous gastric mucosal extracts combined with adjuvants. This model not only recapitulates the characteristic human AIG feature of glandular atrophy specifically in the gastric body/fundus with sparing of the antrum, but also demonstrates late-stage pathological changes such as ECL cell hyperplasia, although its underlying molecular mechanisms require further elucidation [79, 80].

The establishment of non-lymphopenic models more closely mirrors the natural disease process. Furthermore, this approach offers greater modeling flexibility. Immunization during the neonatal period exploits the immaturity of Tregs to establish persistent inflammation, thereby avoiding the reversible nature of inflammation often seen in adult models. However, a significant limitation is the lack of standardization in this modeling method. Variables such as antigen dose, adjuvant type, and immunization schedule lack a unified protocol, leading to poor comparability of results across different studies.

## Summary

This review systematically summarizes the immunological mechanisms of AIG, conceptualized as a immune network in which aberrant activation of CD4⁺ T cells serves as the central driving event. The pro-inflammatory responses mediated by Th1/Th17, functional deficiency of Tregs and immune amplification driven by autoantibodies together constitute an interconnected pathogenic network. Although current research has begun to outline this framework, several key mechanistic questions remain unresolved, hindering the development of etiology-targeted therapeutic strategies. Future studies should prioritize the following directions:

First, a systematic elucidation of the complete functional spectrum of humoral immunity in AIG is needed. While autoantibodies are currently regarded primarily as diagnostic markers, their specific subtype profiles, class-switching, and relationship with disease stages remain unclear. It is essential to establish a more systematic and refined antibody repertoire, integrating high-throughput proteomics and other technologies to profile the expression characteristics of various antibody subtypes and their pathophysiological significance, thereby clarifying the direct role of antibodies in gastric mucosal injury and chronic disease progression.

Second, it is necessary to explore whether Tfh participates in the regulation of the local immune microenvironment in AIG. Its distribution, functional status, and interactions with other T-cell subsets in AIG have yet to be elucidated. Future studies could employ single-cell RNA sequencing, multiplex immunofluorescence, and related techniques to investigate the distribution and functional properties of Tfh in the gastric mucosa or peripheral blood of AIG patients, as well as their specific role within the “T-B cell collaborative circuit”, thereby providing new insights into the mechanisms of immune dysregulation in AIG.

Furthermore, the specific mechanisms by which APCs contribute to immune recognition and inflammation initiation in AIG require in-depth clarification. Although existing evidence suggests that dendritic cells, gastric epithelial cells, and innate lymphoid cells may participate in autoantigen presentation. it remains unclear whether their tolerance to self-antigens is disrupted during AIG development and how different APC subsets cooperatively regulate local immune responses. Integrating single-cell multi-omics, spatial transcriptomics, and other advanced approaches could help dissect the activation states of APCs in the AIG gastric mucosa and their interaction networks with immune cells, ultimately revealing the critical role of APCs in breaking immune tolerance and sustaining the inflammatory microenvironment.

To address these mechanistic questions, there is an urgent need to develop experimental systems that more accurately mimic the pathological features of human AIG. Current animal models have limitations in recapitulating T-B cell collaboration, antibody subtype profiles, and cancer risk. Future efforts should promote the establishment of humanized immune system mice or organoid-immune cell coculture systems, enabling validation of the above scientific hypotheses in contexts more closely resembling the human immune milieu, thereby laying a foundation for the development of immunomodulation-based targeted therapies for AIG.

## Data Availability

No datasets were generated or analysed during the current study.
